# Economics and Energy Consumption of Brackish Water Reverse Osmosis Desalination: Innovations and Impacts of Feedwater Quality

**DOI:** 10.3390/membranes11080616

**Published:** 2021-08-12

**Authors:** Jeffrey L. Pearson, Peter R. Michael, Noreddine Ghaffour, Thomas M. Missimer

**Affiliations:** 1 Emergent Technologies Institute, U.A. Whitaker College of Engineering, Florida Gulf Coast University, 16301 Innovation Lane, Fort Myers, FL 33913, USA; jlpearson@eagle5684.fgcu.edu; 2 Electrical Engineering Department, University of South Florida, 4202 East Fowler Avenue, Tampa, FL 33620, USA; prm@usf.edu; 3 Water Desalination and Reuse Center (WDRC), Biological and Environmental Science and Engineering Division (BESE), King Abdullah University of Science and Technology (KAUST), Thuwal 23955-6900, Saudi Arabia; noreddine.ghaffour@kaust.edu.sa

**Keywords:** brackish water reverse osmosis (BWRO) desalination, capital cost of BWRO, operating cost of BWRO, impacts of feedwater quality on cost, impacts of capacity on cost

## Abstract

Brackish water desalination, using the reverse osmosis (BWRO) process, has become common in global regions, where vast reserves of brackish groundwater are found (e.g., the United States, North Africa). A literature survey and detailed analyses of several BWRO facilities in Florida have revealed some interesting and valuable information on the costs and energy use. Depending on the capacity, water quality, and additional scope items, the capital cost (CAPEX) ranges from USD 500 to USD 2947/m^3^ of the capacity (USD 690–USD 4067/m^3^ corrected for inflation to 2020). The highest number was associated with the City of Cape Coral North Plant, Florida, which had an expanded project scope. The general range of the operating cost (OPEX) is USD 0.39 to USD 0.66/m^3^ (cannot be corrected for inflation), for a range of capacities from 10,000 to 70,000 m^3^/d. The feed-water quality, in the range of 2000 to 6000 mg/L of the total dissolved solids, does not significantly impact the OPEX. There is a significant scaling trend, with OPEX cost reducing as plant capacity increases, but there is considerable scatter based on the pre- and post-treatment complexity. Many BWRO facilities operate with long-term increases in the salinity of the feedwater (groundwater), caused by pumping-induced vertical and horizontal migration of the higher salinity water. Any cost and energy increase that is caused by the higher feed water salinity, can be significantly mitigated by using energy recovery, which is not commonly used in BWRO operations. OPEX in BWRO systems is likely to remain relatively constant, based on the limitation on the plant capacity, caused by the brackish water availability at a given site. Seawater reverse osmosis facilities, with a very large capacity, have a lower OPEX compared to the upper range of BWRO, because of capacity scaling, special electrical energy deals, and process design certainty.

## 1. Introduction

Many regions of the world have limited freshwater supplies to meet the combined demands of human consumption, agriculture, and industry [1]. Limitations on the development and use of fresh groundwater resources have led to assessments on the use potential of saline groundwater. Saline groundwater occurs in abundance in many global locations [2]. Brackish water, with total dissolved solids (TDS) less than about 8000 mg/L, is used as a water supply source in many regions, such as North Africa, central Saudi Arabia, Jordan, and others, in combination with desalination using the brackish water reverse osmosis process [3,4].

**Figure 1:**
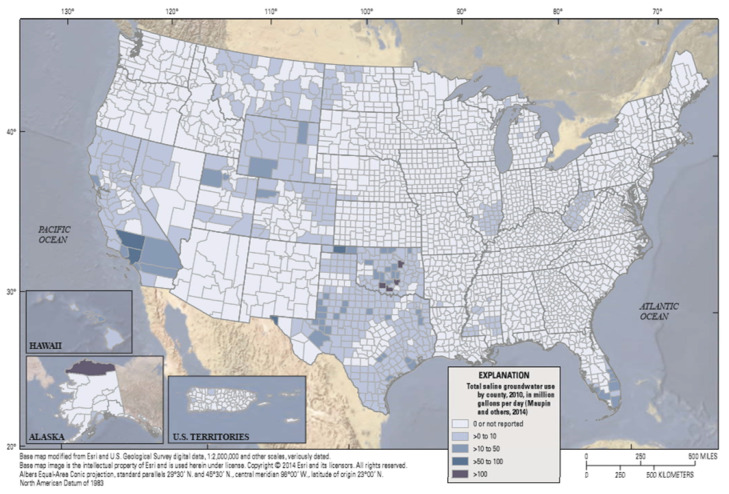
Total saline groundwater use in the United States by county in 2014 [23].

In the United States, there are extensive saline groundwater sources that contain total dissolved solid (TDS) concentrations between 1000 and 10,000 mg/L [5,6,7,8,9,10,11,12,13]. The volume of brackish groundwater that is available for use is quite large and geographically extensive. Saline (brackish) water use by county, in the United States in 2010, is shown in Figure 1. Brackish groundwater is used for irrigation in some regions, when the TDS is under 1500 mg/L, and the vegetation is tolerant to that salinity and the resulting soil salt buildup [14,15,16].

**Figure 2:**
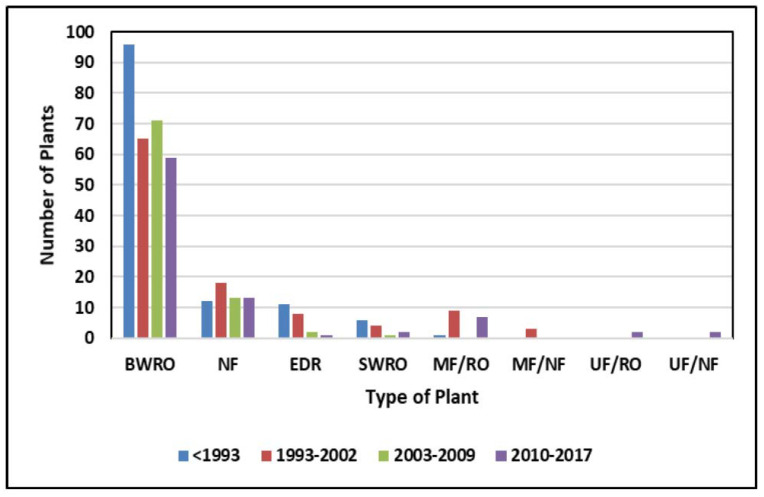
Number of desalination plants operating in the United States in 2010 [19].

Brackish groundwater is also a source of feedwater for many brackish water reverse osmosis (BWRO) desalination plants that produce potable water [8,12,17,18]. In 2010, nearly 250 municipal membrane treatment facilities operated in the United States, and this grew to 406 in 2018 [18,19] (Figure 2). Of this number, 295 are BWRO facilities with capacities over 95 m^3^/d [16].

**Figure 3:**
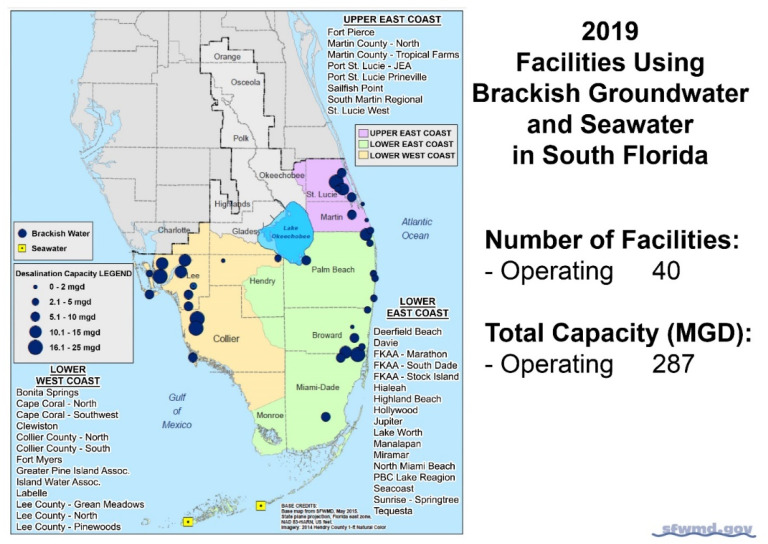
Locations and capacities of operating BWRO desalination plants in Southern Florida [21].

The largest concentration of BWRO desalination plants in the United States occurs in Florida [20,21]. Other states, such as Texas, are planning to add many additional BWRO facilities to those that are currently operating [22]. In Southern Florida, in 2019, 40 BWRO and 3 seawater reverse osmosis desalination plants operated, with a total capacity of 1.09 million m^3^/d [21] (Figure 3).

Because of the global growth rate in the use of brackish water desalination, there is great interest in the capital and operating costs of these facilities, but few data compilations have been published using actual data. One of the purposes of this research is to provide factual data associated with specific facilities and the methods of operation that contribute to the OPEX costs. The costs associated with a total of seven BWRO desalination water treatment facilities, based on cost per cubic meter (m³) in the Southwest Florida region of the United States of America, have been compiled, to allow detailed examination. In Southwest Florida, fresh source water supplies are becoming increasingly hard to develop because of the explosive population growth, impacts on the environment, pumping-induced saltwater intrusion, competition with the use of public or private wells, limited surface-water resources, and climate change issues. The combined results of these factors reduce the available freshwater resources for use and increase the demand for potable water.

In addition to assessing the capital (CAPEX) and operating costs (OPEX) for the Southwest Florida facilities, an evaluation of BWRO costs was made, by comparing these detailed costs with the global data, to construct some basic graphs. These graphs can estimate the unit costs for various capacity plants and the impact of groundwater TDS on these costs. Where possible, the electrical cost was factored into the analysis. Most cost data for BWRO facilities have been estimated, rather than developed from the compilation of actual plant data. The inflation associated with the CAPEX and OPEX of the facilities, has been provided for the US facilities.

Most CAPEX and OPEX cost information on BWRO facilities operating costs is currently estimated using curves, models, or a set of assumptions [24,25,26,27,28,29,30,31,32,33,34,35,36,37,38,39,40,41]. It is a goal of this research to obtain real cost information for the existing BWRO plans, for comparison to the past estimates. Little consideration has been given to innovations in real plant operations, which reduce electrical usage and the overall cost of treatment. Some examples of innovative cost control measures include the blending of raw water with treated water, to raise the pH and to reduce the cost to the consumer; the use of energy recovery devices in BWRO; the blending of the raw water with some limited freshwater, to reduce the salinity of the feedwater (where available); and the implementation of specialized maintenance techniques that extend the lifetime of the membranes, from 5 up to 15 years (e.g., City of Cape Coral).

## 2. Methods

### 2.1. Collection and Analysis of Cost Data

Capital and operating costs were obtained from the oldest, continuously operating BWRO desalination system in the United States, located in the City of Cape Coral, Florida. There are two operating plants in the City with capacities of 68,182 and 45,455 m^3^/d [42]. Data from 5 additional BWRO desalination facilities in Southwest Florida were obtained and compared to the City of Cape Coral facilities. The plants evaluated included the following: the City of Cape Coral Southwest and North BWRO Plants, the City of Fort Myers BWRO Plant, the Lee County Green Meadows and North BWRO Plants, the Pinewoods BWRO Plant, and the Island Water Association BWRO Plant (Figure 3 for locations).

Very detailed data were obtained from the City of Cape Coral plants, which is rare because many utilities, public and private, are reluctant to share financial data. Cost data on the other utilities were less detailed because of the confidential nature of specific data. The monthly operating reports (MORs) for 2017, 2018, and 2019 for each of the seven RO plants were tabulated into Excel spreadsheets based on the information from the Lee County Health Department (Lee County, FL, USA). 

Based on the costs for each of the facilities examined through this research, the level of confidence in the cost data should be reasonably accurate depending on various contracts for chemicals, labor rates, electricity costs, security, etc. Replacement of membranes was not considered in the OPEX analysis for the cost of water per USD/m³ since it could be regarded as either an OPEX or CAPEX item, depending on the timing of membrane replacement within the context of plant expansion. It should also be noted that the Lee County Pinewoods BWRO Plant uses a combination with nanofiltration (NF) and the Green Meadows RO Plant uses a combination of ion exchange (IEX) and BWRO technology that treats both fresh and brackish blended water. Therefore, the Lee County costs per m³ of treated water for these two facilities may artificially appear less than others that use strictly BWRO technology plants. The reason for the combination of technologies was due to the desire by Lee County to continue using fresh groundwater that was already permitted for their NF and IEX plants, along with more recently developed saline groundwater resources that require BWRO treatment. This combined use led to a lower overall cost of potable water to the consumers.

### 2.2. Literature Search on BWRO Costs

BWRO costs were obtained from the literature [43,44], where comparative and useful information could be found. At facilities that contain some unusual components, the impacts of these components are noted in the text. No zero liquid discharge (ZLD) plant costs are not included in this paper, but the technology is discussed.

### 2.3. BWRO Treatment Plant Cost Estimation Methodology 

**Figure 4:**
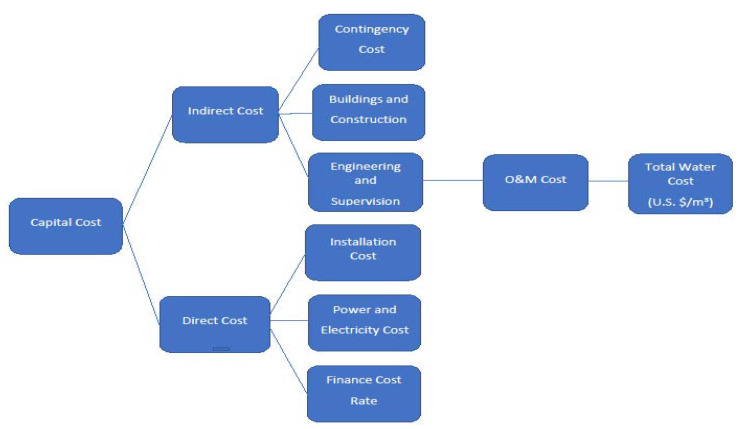
Diagram of total water cost components.

Costs can be classified as CAPEX or investment costs and OPEX, covering operation and maintenance [45]. BWRO plant CAPEX costs typically include land acquisition, all equipment, installation services, design costs, such as civil engineering, mechanical and electrical (M&E) engineering [45]. For most BWRO systems, the O. & M. costs (OPEX) are predominantly controlled by the energy (electricity) and chemical usage in BWRO pretreatment and post-treatment [46], critical component replacement (the BWRO membranes and pumps), water supply, and concentrate disposal charges, and other items, such as labor and servicing [45]. CAPEX and OPEX can be combined to produce the net present value (NPV), which accounts for the cost of financing by assuming that investment of the capital sum elsewhere will produce an annual return quantified by the discount rate [45]. A flow diagram for cost determination is shown in Figure 4.

## 3. Background

### 3.1. Description of Facilities in Southwest Florida

#### 3.1.1. City of Cape Coral Southwest BWRO Plant

The city currently operates and maintains two reverse osmosis (RO) water production facilities, 55 raw water production wells, a raw water transmission system, two water storage and re-pump stations, and a potable water transmission and distribution system. The Southwest Water Treatment Plant (WTP) is the oldest, continuously operated brackish low-pressure BWRO potable water production facility globally. The Southwest WTP has two separate BWRO WTPs in one facility that operate independently. Plant 1 has a design capacity of 22,710 m³/day. Plant 2 is the larger unit, with a design capacity of 45,799 m³/day. Together, the plants combine for a total design capacity of 68,509 m³/day. Both of the plants utilize the same process, which consists of chemical pretreatment, micron (cartridge) filtration, reverse osmosis, degasification, pH adjustment, and chlorination. The concentrate (brine) from both the plants is disposed of via deep-well injection into the boulder zone, located approximately 914.6 m below the land surface (BLS).

#### 3.1.2. City of Cape Coral North BWRO Plant

The North BWRO WTP was placed into service on 8 March 2010, and has a permitted production capacity of 45,420 m³/day, which can be achieved by using approximately 54,882 m³/day of raw water, while producing approximately 9462 m³/day of concentrate that requires disposal (approximate 83% recovery). The firm capacity of the North WTP (with one unit out of service) is 34,065 m³/day. The North WTP utilizes the BWRO process. 

The BWRO membrane elements are low-pressure, high-rejection thin-film composite membranes. Pretreatment of the raw water from the wellfield is through a combination of sulfuric acid, polyacrylic acid, and filtration. This form of pretreatment has been used very successful at the Southwest WTP, to meet water quality performance standards at a reasonable treatment cost and extends the life of the membrane elements. Following pretreatment, the water travels under pressure to each of the four treatment trains. Each treatment train has a production capacity of 9462 m³/day. A 298,280 watt (≈400 hp) pump then conveys the water into the first stage of the treatment train, to begin the RO process, which has two stages. The product water from the two-stage RO process is combined and blended with raw water, to meet the target water-quality parameters. 

Disposal of the concentrate (brine) is through a single on-site deep injection well (IW). The use of blended product water is cost-effective, as it reduces the amount of post-treatment chemicals that are needed for controlling the hardness and alkalinity. The blended water is then post-treated for the removal of gases (H_2_S). After traveling through the degasification process, the blended water enters a 643.45 m³ capacity clearwell. In the clearwell, sodium hydroxide is added to raise the pH to stabilize the water, and sodium hypochlorite is added for disinfection. The water is pumped to the 45,420 m³ pre-stressed concrete ground storage tank, using one of the two 75,600 watt (≈100 hp) transfer pumps. Both the City BWRO Plants are supplied with brackish raw water from the Lower Hawthorn Aquifer, located in the upper part of the Floridan Aquifer System. There is a total of 55 raw water production wells.

#### 3.1.3. Island Water Association BWRO Plant

The Island Water Association, Inc. (IWA), one of the oldest BWRO systems in the world, began its operation in 1982. It is a member-owned corporation that supplies water to a franchise area covering the City of Sanibel (Sanibel Island) and Captiva Island, Florida. The BWRO plant has an installed capacity of 22,331 m^3^/d, including a blend of raw water. It can produce a maximum of 26,477 m^3^/d [47]. The design of the plant includes thin-film composite membranes in a two-stage configuration. It can treat a TDS concentration of up to 3500 mg/L. The average current TDS concentration of the feed water is 2800 mg/L [47]. 

#### 3.1.4. Lee County Green Meadows BWRO Plant

The Green Meadows water treatment complex contains both freshwater treatment and a BWRO facility. It is located in east-central Lee County, Florida. The facility serves approximately 20,000 homes and businesses in Lee County, as one of the key water supply facilities. Originally built in 1977, the wellfield consisted of a 34,091 m^3^/d capacity lime softening treatment plant, and 27 water tables and Sandstone aquifer production wells (freshwater) running along an 8 km unpaved access road before the expansion. The upgrades turned the 40-year-old WTP into a state-of-the-art facility, converting the treatment process to combined ion exchange and BWRO, and increasing the finished water production capacity to 53,030 m^3^/d. The wellfield expansion added eight upper Floridan Aquifer System production wells (brackish water) and a deep-well concentrate disposal system, consisting of an 876 m deep injection well and a 549 m deep dual zone monitoring well. The Lee County Green Meadows BWRO Plant was placed into operation in May 2018. The plant has a permitted capacity of 60,560 m³/day. The plant utilizes a combination of ion exchange to treat the freshwater and BWRO to treat the brackish raw water [48]. 

#### 3.1.5. Lee County North BWRO Plant

The Lee County North BWRO Plant has a permitted capacity of 43,906 m³/day [49]. The plant was constructed in 2006 and treats brackish groundwater from wells that are drilled into the Lower Hawthorn Aquifer, which is the uppermost aquifer in the Florida Aquifer System [50]. The plant disposes of RO concentrate via a deep injection well. 

#### 3.1.6. Lee County Pinewoods NF/BWRO Plant

The Lee County Green Pinewoods BWRO Plant was originally placed into service in 1990, as a nanofiltration (NF) plant. Lee County Utilities took over plant operation of the 8705 m³/day capacity facility in 2003. Raw water is obtained from wells that are drilled into the water table and Sandstone aquifers (freshwater). The plant was upgraded in 2005–2006, by adding an 11,355 m³/day BWRO plant, with wells drilled into the Lower Hawthorn Aquifer. The facility has a current permitted capacity of 20,060.5 m³/day. A combination of nanofiltration (NF) and RO, to treat the raw water from the freshwater and brackish water wells, is used, respectively [51].

#### 3.1.7. The City of Marco Island South BWRO Treatment Plant 

The City of Marco Island operates a BWRO plant with a capacity of 22,710 m³/day. Feedwater is pumped from a series of 18 upper Florida Aquifer System wells that provide between 30,280 and 31,037 m³/day of brackish water [52]. The feedwater is rough-filtered to remove any potential particulate material. The feedwater is pretreated with a scale inhibitor to prevent scaling, and sulfuric acid to reduce the pH. The raw water then travels through six cartridge filters to remove very fine particulate materials, and is then pumped at high pressure and sent into the six dual-stage BWRO membrane trains. The reject water is pumped to the Marco Island Wastewater Plant for injection into a deep disposal well (976 m). The permeate from the membranes is treated with sodium hypochlorite and ammonia, to form chloramines, and then mixed with air in one of the two degasifiers that are used to remove hydrogen sulfide. The water is conveyed into the storage tanks, and the stripped gases from the degasifiers go into an air scrubber to remove the hydrogen sulfide from the air, before it is dispersed into the atmosphere.

## 4. Results

### 4.1. City of Cape Coral North Plant Capital Cost Analysis

It is quite challenging to assess the comparisons of CAPEX for BWRO facilities, because of the differences in the overall project objectives, the design feedwater quality, and the project delivery (e.g., design-bid build, build-own-operate-transfer). CAPEX is highly location-specific, based on multiple project objectives that may include distribution system improvements, varying capacities of surface storage, provisions of the plant footprint to accommodate expansion, the method of concentrate disposal, and differences in post treatment.

The City of Cape Coral North Plant is a prime example of the complexity of a large-scale BWRO project. This dataset is unusual in that it is detailed and can be used to obtain a variety of different and useful pieces of information. The base capacity for the plant was 45,420 m³/day. When the entire facility cost of USD 138.5 million is divided by the capacity, the resulting cost per installed m^3^ is USD 2947, which must be considered a very high number. Therefore, it is essential to assess the details of the project, as shown in Table 1. 

The high CAPEX per m³ is a result of the desire to size the building to accommodate additional treatment trains and raw water wells, to treat a maximum plant capacity of 113,550 m³/day of water. In addition, the pipelines from the plant were designed to accommodate the buildout capacity. Therefore, the CAPEX cost per m³ of water would be considerably lower than the USD 2947/m^3^. Also, the land area acquired was larger than that required for the initial plant capacity, and it has some other planned uses. If the total project cost was divided by the ultimate plant capacity, the unit cost would be USD 1215/m^3^. However, the equipment cost for the increased capacity would also have to be added back. Therefore, the actual unit cost would be about USD 1800/m^3^. A key issue is the high cost of the land purchased for the utility “campus”, which will include other infrastructure, not only the BWRO facility.

Capital costs are not commonly reported, but in the cases where reports have been made, they are inconsistent, incomplete, and contain minimal details. The delineation between civil and material costs can differ between reports, as can the identification of critical component replacement items. The membrane costs for replacement are also inconsistently reported, because they can be related to both CAPEX and OPEX. For example, some facilities purchase a certain number of replacement parts as part of the CAPEX. Additional spare components can include high-pressure pumps, values, and others that have a long back-order time. Some plants that expect significant increases in feedwater salinity may also over-size the horsepower of the high-pressure pumps, to accommodate the future salinity changes (de-staged pumps). 

If only the initial capacity of the City of Cape Coral North Plant and those associated constructed costs are considered, the estimated cost per installed m^3^ would likely range from USD 700 to USD 1000. The land cost is particularly high at this location, because the site acquired contained platted building lots in the middle of a residential area. The engineering and design for the BWRO plant were incorporated within a large-scale utilities program management scheme, with an engineering firm taking the role of the program manager. The CAPEX for the other six plants could not be obtained in sufficient detail to be reported.

### 4.2. Cost of Water for Southwest Florida BWRO and Hybrid Facilities (OPEX)

**Figure 5:**
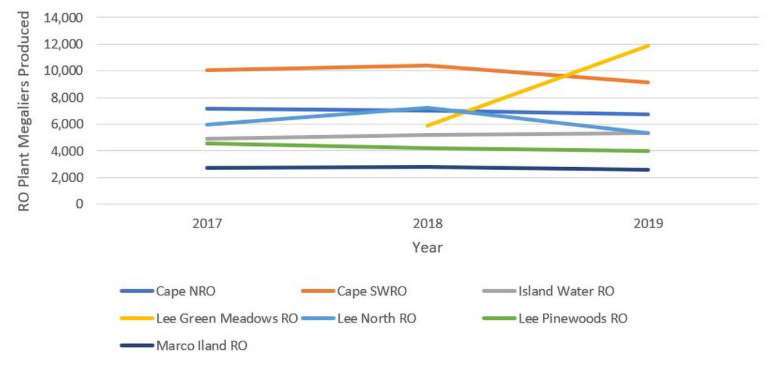
Produced water from seven BWRO plants in Southwest Florida in megaliters from 2017 to 2019. Note that the Lee Green Meadows RO Plant began operating in May 2018; no data were available for 2017 and January–April 2018.

**Figure 6:**
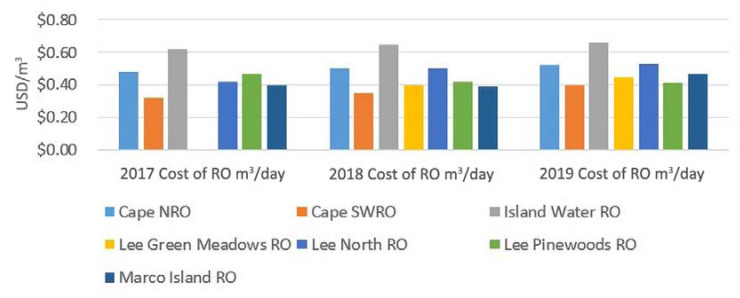
Southwest Florida RO desalination plant cost of water produced in (USD/m³). Note: the Lee County Green Meadows RO Plant began operating in May 2018; therefore, no data were available for 2017 and January–April 2018. Note that the consumer price index for the 3 years was 6.3% or slightly over 2%/year.

The estimated OPEX costs for seven BWRO facilities (including hybrid systems) were evaluated over 3 years, from 2017 to 2019. The produced water volume from these facilities is summarized in Figure 5, and the estimated OPEX cost/m^3^ is given in Figure 6.

Some significant facts can be obtained from the data. OPEX, from year to year, is not constant, and there is a general upward trend in the studied facilities. The average increase in OPEX cost, between 2017 and 2019 (3 years), is 4.87%, with the range in plant changes from 2 to 6.9%. The total consumer price index rise over the 3-year timeframe was only 6.3%, so the operational cost change was lower than this number. There is some scale to the costs of the pure BWRO systems, with the Island Water Association having a higher cost. Marco Island is another low-capacity plant, but may have reduced cost due to a blend of freshwater taken from a lake source. The Cape Coral plants, the Lee County North plant, and the Island Water Association plants have purity in BWRO operation, without any hybrid treatment schemes. The average TDS of the feedwater at these facilities does not seem to be a significant OPEX factor (Table 2).

The OPEX cost of brackish water RO drinking water in Southwest Florida ranges between USD 0.36/m³ and USD 0.66/m³, as reflected in Table 1 and Table 2. A comparison between the pure BWRO plants only includes the two City of Cape Coral plants, the Island Water Association plant, and the Macro Island plant. However, the range in OPEX numbers does not change.

### 4.3. Cost Analysis of Various BWRO Plants in Texas 

**Figure 7:**
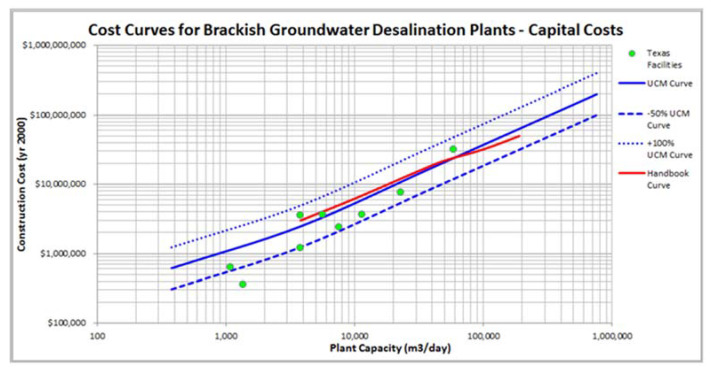
Comparison of the CAPEX costs for nine BWRO plants in Texas to the cost curves developed by Unified Costing Model [53] and the Desalting Handbook for Planners [33]. This cost model has not been corrected for the consumer cost increase from when the model was created to the current cost.

**Figure 8:**
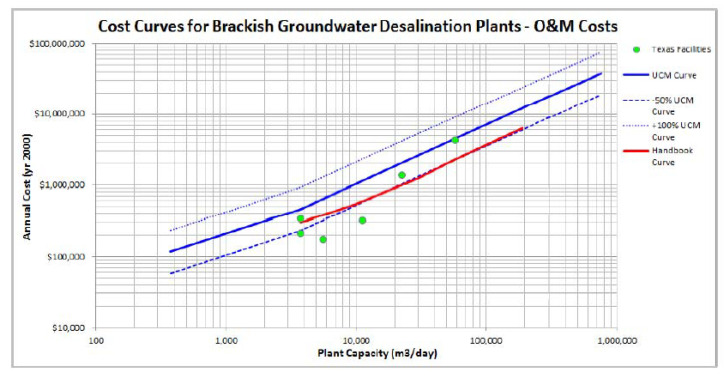
Comparison of the OPEX costs for nine BWRO plants in Texas to the cost curves developed by the Unified Costing Model [53] and the Desalting Handbook for Planners [30]. Note the cost increase may have occurred at these utilities since the data were compiled for use in the model until current conditions.

Cost data are available from several BWRO plants in Texas [43,44]. A summary of the data is presented in Table 3. The range in capital cost is 536 to 1032 USD/m^3^ [39]. This range is somewhat misleading, in that the blended cost reduces this range, and there are data from one facility that seem to be too low. The OPEX cost range in USD is 0.23 to 0.63/m^3^. Two useful figures were produced by the USBOR [40] that show cost curves for CAPEX and OPEX, with comparisons to some published cost curves (Figure 7 and Figure 8). A key observation on the Texas electric cost data is comparatively low, at USD 0.059 to 0.083/kWh. 

### 4.4. A Compilation of Various International BWRO Plant Capacities, Feed-Water Quality, and Treatment (OPEX) Cost/m^3^

A compilation of data on international BWRO facilities, with capacities ranging from <20 to 60,000 m^3^/d, is given in Table 4. The very-low-capacity facilities have the highest unit costs for desalination, as would be expected. However, the relationship between the capacity and unit cost is not uniform once a capacity of 1200 m^3^/d is exceeded. The TDS of the water, electrical rate, and pretreatment costs must all impact the cost to some degree. However, the range in costs, between 0.19 and 0.48 USD/m^3^, is relatively narrow.

## 5. Discussion

### 5.1. CAPEX Cost Variation

The CAPEX costs for BWRO facilities vary in the extreme, based on what is included in the project and land costs. The highest cost documented value of over USD 2947/m^3^ is the North City of Cape Coral, Florida facility. Based on the consumer price index over the years, since the construction of the project, the 2020 cost would be about USD 4067. The detailed breakdown of this cost shows that a large urban land purchase was required to build the BWRO facility, and to accommodate other utility infrastructure at the same location. The building was sized to meet the buildout capacity for the service area of the plant. Also, spare pumps were purchased in addition to other equipment that was needed to provide operational security. The raw water contained hydrogen sulfide, which had to be removed before the treated water could be discharged into the distribution system, so a degassing system had to be designed and constructed. The concentrate disposal for the plant was a deep-well injection, so that the cost was also included in the CAPEX. The Cape Coral North facility is an excellent example of why using cost curves to estimate CAPEX, for new BWRO facilities, can produce inaccurate estimates. Many projects involving BWRO design and construction also include multiple components that cannot be easily separated from the primary project goal. Commonly, distribution system improvements are also contained with these project budgets (e.g., storage tanks, pumping stations, and pipeline improvements). The use of cost curves for the estimation of strictly the BWRO component of a project is reasonable, if it can be separated from the other components of the project.

Perhaps the most consistent data set on BWRO CAPEX is that from Texas. These projects had a rather narrow set of goals and objectives that follow the general trend of reducing CAPEX/m^3^ with increasing plant capacity. The one possible exception is the largest capacity plant in El Paso (Figure 7). This facility uses a high-pressure deep injection well system for concentrate disposal.

### 5.2. OPEX Cost Variation

Some important general observations can be made in analyzing the complied OPEX data from various BWRO facilities. First, the local electrical cost is one of the key factors that controls BWRO treatment costs in facilities that treat raw water, within a TDS range of 2000 to 8000 mg/L, and do not have major pretreatment and concentrate disposal challenges. The OPEX costs for BWRO facilities in Florida are generally higher than in Texas, because the average electrical cost is about USD 0.6/kWh in Texas versus USD 0.125/kWh in Florida. The Florida rate is primarily for residential electric use, and industrial or utility rates are lower based on the negotiated rates, considering factors such as interruptible power (on-site generators) and peak load reduction. Most BWRO facilities in Florida must also post-treat the potable water to remove hydrogen sulfide. The international facility costs are lower, which could result from various types of subsidies, or other unknown reasons. At locations where the pretreatment costs only include an antiscalant and acid to reduce the pH, the costs are generally lower. Where any additional control of the substances in the raw water is required, such as silica, iron, or manganese, the costs can be quite high compared to the pretreatment that is used to solely control calcium carbonate scaling. 

**Figure 9:**
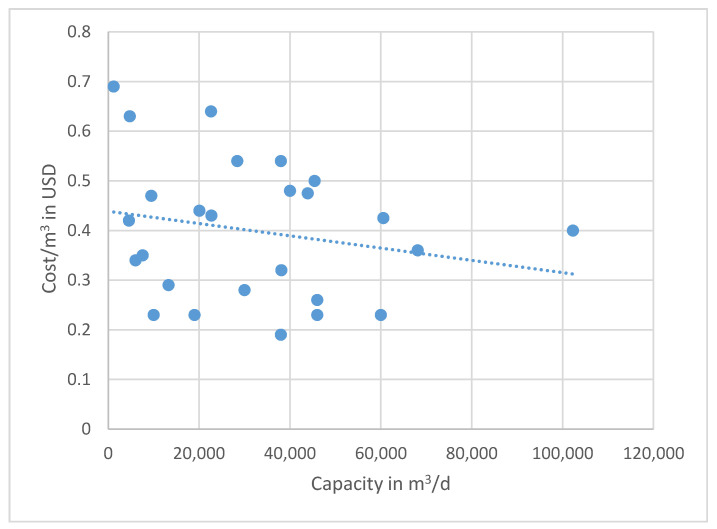
U.S. and global OPEX cost for various plant capacities. Note the extreme scatter of data based on the arithmetic plot. The R^2^ value of the trend line is poor, with the data having a standard deviation of 0.13 m^2^/d, but shows a reducing unit cost as capacity increases. Note that these costs are not corrected for local inflation, which may not directly correlate with increased costs to consumers (e.g., foreign water subsidies).

**Figure 10:**
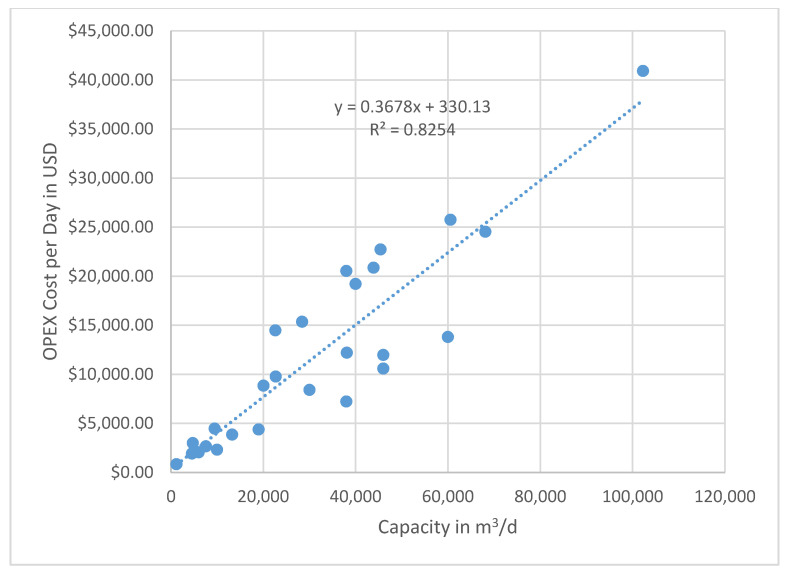
Plot of capacity versus total OPEX cost/day. Note that this plot shows a better fit to the trend line with a high R^2^ value. The equation for the trend line has a slope of less than one-to-one, which is indicative of a lower unit cost with a larger capacity. Therefore, scaling of capacity is an important economic factor. Note that these costs are not corrected for local inflation, which may not directly correlate with increased costs to consumers (e.g., foreign water subsidies).

In general, the cost for BWRO plants capacities, ranging from 10,000 to 70,000 m^3^/d, is 0.39 to 0.66 USD/m^3^ based on the time when the data were collected. These data are not corrected for inflation, because the individual utilities do not raise the consumer rates annually and may lag behind real cost recovery. A graph showing all of the compiled OPEX data based on plant capacity (m^3^/d) versus cost in USD/m^3^ is given in Figure 9. Note that there is extreme scatter when plotting all of the data that were collected. The variations are caused primarily by the differences in energy costs, pretreatment of the feedwater, and post-treatment of the finished water. There is no distinct pattern of reducing unit costs with increasing plant capacity for all of the data combined. Still, the trend line shows a negative slope, indicating a reduction in OPEX as the capacity increases. The Texas data show a tighter fit to the capacity versus cost scaling line. A second plot of the plant capacity in m^3^/d versus the cost per day in USD produces less scatter, and a trend line can be drawn with a better correlation (Figure 10). Since the slope of the line is less than one-to-one, as shown in the regression equation, the scaling factor that indicates a lower unit cost as the capacity increase is again confirmed.

### 5.3. Impacts of Energy Recovery Systems in BWRO

In the past, energy recovery systems were not considered to be effective in cost savings in BWRO plants, because of the low operating pressure, high recovery, and the use of blending with raw water. However, many BWRO plants are now being designed with energy recovery systems or retrofitted with these systems.

An evaluation of two types of energy recovery systems for a BWRO plant showed that both turbocharger and isobaric systems might save electric energy costs, especially when the conversion rate is <84% [57,58,59]; as the conversion rate declines, the electrical energy saving increases [60]. The key method in deciding on the use of energy recovery systems on BWRO plants is to conduct a complete life cycle analysis (LCA) [61].

A large number of BWRO facilities exhibit long-term increases in feed water salinity [62,63,64,65,66]. The use of energy recovery systems to mitigate electric energy costs increases as the feedwater salinity increases, which should be considered. This would potentially mitigate higher energy consumption, to treat higher TDS feedwater. Some recent research on the use of energy recovery in BWRO has suggested integrating a supercapacitor with capacitive deionization into the process [67].

### 5.4. Impacts of Feedwater Chemistry Issues That Can Potentially Affect the Economics of Brackish-Water Desalination

Feedwater chemistry can significantly impact BWRO costs if additional pretreatment is required to prevent membrane scaling and fouling [68]. In BWRO, the most common problem is scaling and not fouling, although some fouling has been reported at the City of Cape Coral North BWRO facility. There are four common types of scaling in BWRO plants, which are impacted by the feedwater chemistry. This includes scaling with calcium carbonate, calcium sulfate, iron, and silica [69].

Calcium carbonate scaling is commonly controlled using standard pretreatment methods, as described in this paper at the City of Cape Coral facilities. The pH of the inflow water is lowered using acid, and a polyphosphate or polyacrylate is added. Some recent research has been conducted on the use of polyaspartic acid as a pretreatment additive [70]. Unless the hardness of the feedwater is exceptionally high, the standard pretreatment process that is used to control calcium carbonate scaling does not add a higher cost to BWRO desalination. In feedwater with a very high hardness, the additional concentration of acid required could increase OPEX cost.

The control of calcium sulfate scaling (gypsum) can be considerably more complex [71,72]. The closer to saturation that occurs in the feedwater, the greater the difficulty of the pretreatment. A zwitterionic coating on the thin-film composite membranes has been suggested as a means for slowing gypsum scaling [73]. The addition of carboxymethyl cellulose in the feed may also reduce the rate of scaling [74]. The use of sulfuric acid to lower the pH is not recommended, but hydrochloric acid is more effective and does not add additional sulfate to the feedwater. However, hydrochloric acid is more expensive and tends to increase the treatment costs. When the feedwater chemistry contains a high relative concentration of sulfate-to-chloride ratio, the potential for scaling rises and increases the potential for gypsum scaling, the recovery using the RO process must be reduced, thereby significantly increasing the treatment cost [75]. In certain cases, it is more effective to treat this type of feedwater using electrodialysis or electrodialysis reversal if the overall TDS concentration is not too high [76,77,78]. In Florida, this issue occurs in Sarasota County.

The control of iron scaling in BWRO is commonly mitigated by the reduction in the feedwater pH, similar to calcium carbonate. However, if the dissolved iron concentration is too high or the feedwater varies between anoxic and oxic, the iron must be removed at considerable expense. In this case, it may be necessary to add a tray aerator, add a coagulant polymer, and then use a plate settler to remove the iron [79]. An alternative would be to use chlorine dioxide, a plate settler, micro-sand filtration, and then an oxidant remove stage to remove the iron [79]. Therefore, if the dissolved iron must be removed, the BWRO desalination process rises in cost.

Perhaps the most difficult potential scaling issue is that with silica. Most natural groundwater sources do not have high silica concentrations, but in aquifers with elevated temperatures (geothermal), silica concentrations can approach saturation or they can contain silica colloids. Because the scaling of silica on BWRO membranes may not be able to be removed, it is quite important that it be prevented from occurring. Two factors seem to dictate the scaling of silica on the membrane, which include the initial concentration in the feedwater (combined dissolved and colloids) and the surface condition of the membranes [80,81]. The surface electrostatic charge and the occurrence of certain organic materials can either accelerate or inhibit silica deposition. The typical pretreatment methods to prevent scaling are pH adjustment or to add an antiscalant solution [82,83]. These methods are common and do not generally add significant costs to the pretreatment. However, if pretreatment of the feedwater is required, then the costs can become quite high. Expensive pretreatment techniques, such as electrocoagulation, can be used to remove the silica from the brackish water before primary membrane treatment [84].

### 5.5. Impacts of Zero Liquid Discharge on the Economics of BWRO

Disposal of the concentrate after the BWRO process has also become a potentially large cost factor within the interior facility locations, where surface disposal into the ocean or the use of deep injection wells is not possible. In some locations, the use of zero liquid discharge (ZLD) is the only means of concentrate disposal. Within the realm of seawater desalination, the use of ZDL for large-capacity plants is likely a myth [85]. In small- to medium-capacity BWRO systems, ZLD is feasible, but requires a means of salt disposal, which causes increased costs for both the additional energy for treatment and the solid waste disposal [86,87,88]. A number of methods have been proposed to lower the energy consumption and costs for ZLD, by combining various membrane and thermal processes [89,90,91,92,93,94]. All of the methods, either used or designed to date, cause a major increase in power consumption, resulting in higher water production costs [95]. Perhaps mitigation methods could be used to co-locate inland BWRO plants, where a number of industries require very-high-quality water for makeup water to produce steam or where valuable metals could be extracted from the waste stream. 

### 5.6. Comparative Costs between BRWO and Seawater RO (SWRO) Costs

Over the past decade, the cost of SWRO has steadily decreased, based on the common use of energy recovery devices and the scale factor that is associated with the design and construction of very-large-capacity facilities [38]. The OPEX cost of some seawater plants has now fallen below BWRO OPEX costs for some of the larger facilities, in the 100,000 to 1,000,000 m^3^/d range. A key difference between the SWRO and BWRO facilities is the chemistry differences in the raw water supply, and the ability to design and construct very-large-capacity SWRO plants above 200,000 m^3^/d. The issue of concentrate disposal is another factor favoring a lower seawater desalination cost, where the disposal is back into the ocean. While equivalent-capacity plants will continue to show higher OPEX costs for BWRO, the trend will continue to reduce SWRO costs as the capacities grow. It is interesting to note that the use of seawater from groundwater sources should significantly reduce pretreatment, due to the lack of organic carbon in groundwater. However, the possible occurrence of hydrogen sulfide in the feedwater will necessitate its removal after membrane treatment, and thereby could cause an increase in the overall treatment cost.

## 6. Conclusions

An analysis of the actual BRWO CAPEX costs illustrates the vast variability based on the scope of the facility design. Commonly, the actual BWRO plant construction is only part of an overall project and cannot be easily separated. Other utility system improvements, spare pumps and membranes, specialized concentrate disposal systems, and other costs, are added to the overall CAPEX. Specialized pretreatment equipment to remove silica, iron, and manganese, and post-treatment equipment to remove hydrogen sulfide, can also cause CAPEX variability. A “normal” range in CAPEX is roughly USD 500–USD 600/m^3^ (USD 550–USD 660 with inflation correction). However, much greater costs can occur, such as the City of Cape Coral North Plant, which had a cost of over USD 2974/m^3^ (over USD 4000 with inflation), because of the various scope items funded under the project.

OPEX costs also vary considerably based on the site-specific water chemistry, electricity costs, and, to a degree, the scale factor that is associated with the plant capacity. One surprising observation is that in the feedwater TDS range of 2000 to 6000 mg/L, there is little difference in OPEX based on the low pressure of the membrane operation and the improvements made in the BWRO membrane design over the last few decades. However, there is a distinct scale trend with the unit cost reducing with increased capacity, as expected. Major OPEX difference increases occur in zero liquid discharge systems, at facilities located inland, away from the sea, or areas where inexpensive deep-well injection disposal is not an option.

Many BWRO facilities use groundwater for feed. In many locations, the groundwater tends to increase in salinity with time, as pumping induces upward and/or lateral migration of the higher salinity water. In the past, such changes in salinity would result in higher energy consumption to treat the water, but energy recovery systems may be used in the future to mitigate the increased salinity. However, once the TDS reaches some critical concentration, there will be a necessity to change the membrane to a higher-pressure type, with an inherent lower recovery and greater energy consumption. Then, the costs will rise eventually towards the SWRO costs.

## Figures and Tables

**Table 1:** Total CAPEX costs for the City of Cape Coral North RO Plant were constructed beginning in 2006 and completed in 2010. The initial capacity of the plant was 45,420 m³/day, with the facility designed to be expanded to 113,550 m³/day.

Work Authorization	North Cape Coral BWRO Facility Costs (USD)
Site Master Plan	449,500
Site Common Facilities Design	317,736
Permitting	241,647
North RO Water Treatment Plant Design	3,566,654
North RO Water Treatment Plant Site and Civil	6,071,679
North RO Plant Construction	92,804,241
North RO Deep-Well Injection Design	131,552
North RO Deep Injection Well Construction	9,556,963
North RO Wellfield Design	439,417
North RO Wellhead and Generator Design	809,482
North RO Wells and Generator Construction	6,022,448
North RO Raw Water Transmission Design	1,640,916
North RO Central Loop Raw Water Transmission Construction	2,525,476
**Summary**	
Total Design, Planning and Construction Cost	124,577,711
Total Land Cost	4,601,466
Total Program Management Cost	4,668,183
Total North RO Plant Cost	133,847,360

**Table 2:** Comparison of capacities, feedwater TDS, range in OPEX over 3 years, and average cost over 3 years. Note that the Lee County Pinewoods Plant has less than 3 years of data. Note that the general increase in cost correlates well with the annual rate of inflation of 2%/year.

Plant	Capacity (m^3^/d)	Avg. Feedwater TDS (mg/L)	Range in OPEX (USD/m^3^)	Average OPEX (USD/m^3^)
City of Cape Coral North	45,420	2452	0.48–0.52	0.50
City of Cape Coral Southwest	68,130	2132	0.32–0.40	0.36
Island Water Association	22,617	2800	0.62–0.66	0.64
Lee County Green Meadows IX & RO	60,560	2913	0.40–0.45	0.425
Lee County North	43,906	None Reported	0.42–0.53	0.475
Lee County Pinewoods RO & NF	20,060	3848	0.41–0.47	0.44
Marco Island	22,710	None Reported	0.39–0.47	0.43

**Table 3:** BWRO plants in Texas USA (recalculated from Arroyo and Shirazi [43], original costs in year constructed). Note that the compiled consumer cost index increase from 2002 to 2020 is about 38% or close to 2%/year in the US.

Plant Name	Year Built	BWRO Capacity (m^3^/d)	Capacity with Blend (m^3^/d)	Feed Water TDS (mg/L)	Pretreatment	Post Treatment	Total Cost (USD × 10^6^)	Cost USD/m^3^ without BLEND	Cost USD/m^3^ with Blend	OPEX (USD/m^3^)	Power Cost USD/kWh
NAWSC Victoria	2012	7576	8523	3800	NA	NA	3.7(?)	488(?)	434(?)	-	-
NAWSC Doolittle	2008	11,364	13,258	2500–3000	CF, CA	Gas removal, pH adj., DI	8	704	603	0.29	0.069
NAWSC Owassa	2008	5682	7576	2500–3000	CF	Gas removal, pH adj.	5.85	1030	772	0.35	0.059
Fort Hancock WCID	2012	1894	NA	2000–2400	CF	NA	3.375	1782	NA	0.86	0.082
Roscoe	2013	1364	1894	3800	NA	NA	0.974	714	514	0.23	0.07
Kay Bailey Hutchinson	2007	56,818	102,272	2000–3000	CF, scaling control	pH adj., corrosion control, DI	91	1602	890	0.40	0.0835
North Cameron Regional	2007	3788	4735	3500	CF, CA	Gas removal, pH adj., DI	7	1848	1478	0.63	0.08
North Cameron Regional	2007	7576	9470	3500	CF, CA	Gas removal, pH adj., DI	8	1056	845	0.47	0.08
Southmost	2004	22,727	28,409	3500	CF, CA, Antiscalant	Gas removal, pH adj., CC, DI	23	1012	810	0.54	0.0749
NAWSC Lasara	2005	3788	4545	2500–3000	CF, CA	Gas removal, pH adj., DI	1	528	440	0.63	0.072
North Alamo WSC Donna	2012	9470	NA	3800	NA	NA	6.7	707	NA	0.37	0.7

**Table 4:** OPEX cost data for various BWRO plants. Uncorrected for local inflation costs since construction.

Plant Capacity (m^3^/d)	Total Dissolved Solids Concentration (mg/L)	Cost (USD/m^3^)	Source
<20	-	5.08–11.55	Atab [51]
50	5700	7.24	Voutchkov [52]
20–1200	-	0.69–1.19	Atab [51]
6000	8116	0.34	Wilf [53]
10,000	4221	0.23	Wilf [53]
19,000	-	0.23	Atab [51]
30,000	5844–11,688	0.28	Wilf [53]
38,000	-	0.19	Atab [51]
~38,000	10,000	0.54	Al-Karaghouli [54]
~38,000	3000	0.32	Al-Karaghouli [54]
40,000–46,000	-	0.23–0.48	Atab [51]
46,000	5000	0.26	Zarzo [55]
5000–60,000	-	0.23–0.48	Chandhry [56]

## Data Availability

Not applicable.
